# The Use of Alcohol in Medical Practice

**Published:** 1874-06

**Authors:** John Morris

**Affiliations:** Baltimore.


### ARTICLE II.

### *The Use of Alcohol in Medical Practice.*

BY JOHN MORRIS, M. D., OF BALTIMORE.

A report read before the Medical and Chirurgical Faculty of Maryland.

The medicinal use of Alcohol may be truly said to be the growth of this century, and, as it is charged that the employment under the sanction of the profession has wrought much injury to the public good, we think it is fitting that the matter should be thoroughly investigated. It is also asserted in many quarters, that alcohol is not a necessary means of cure, that it is only an excitant and not directly a life sustaining force, and that as we have so many other remedies at our command equally potent in medicinal power, and not absolutely injurious in their remote consequences, it would seem to be wise to exclude it as an agent in the cure of disease.

To examine the truth or error of these statements is the purpose of this paper, and to this end we shall investigate the following points:

First—Is alcohol food?

Second—Is alcohol a poison?

Third—Is it absolutely necessary in the treatment of disease?

Fourth—Is the good it works in the cure of disease at all equal to the amount of disease it produces?

Fifth—How far are medical men responsible for its use as a dietetic agent?

Sixth—What can the medical profession do by example and teaching to restrict its use.

In our consideration of these questions, or at least a portion of them, it will be necessary to examine the physiological action and therapeutic effects of alcohol, as well as the pathological changes brought about by its use.

First, then, as to its physiological action. This has been made the subject of the severest investigation in the past few years, in the hands of such well-known men as Parkes, Anstie, Thudicum, Dupre, Richardson, and lately Subbotin. These gentlemen differ very widely in their conclusions.

Whilst Anstie and Dupre claim that no elimination of alcohol takes place from the body, but that it remains in the system as a life-supporting, life-giving force, Parkes, Subbotin and others, as strenously assert that it is in great part eliminated, and what is left is only a source of injury to the organism. A fair judgment based on a careful examination of all the authorities on the subject is, that a certain amount of alcohol is eliminated from the body by the lungs, skin, kidneys, etc., but that a large quantity remains in the system in a state of oxidation. Doctor Anstie, the boldest, most zealous, and perhaps the most logical of the writers who contend that the ingestion of alcohol and its chemical destruction within the body give rise to useful force, argues that alcohol is a hydro-carbonaceous substance, which can be taken into the organism to the extent of one and a half ounces without exciting morbid phenomena, and that like other hydro-carbonaceous substances taken into the system, it is devoted either to the construction of tissue, or to the generation of force, which will display itself obviously under some one of the modes of motion. On the other hand, Parkes, Subbotin and others, deny this, and believe it plays no part in the formation of tissue. Subbotin says that under the influence of alcohol, metamorphosis of tissue diminishes, the temperature is lowered, there is a smaller amount of carbonic acid excreted, and also a smaller quantity of urea found in the urine. He thinks it acts immediately upon the nervous system, and can only be classed as an excitant. The physiological action of the heavy alcohols has been made the subject of investigation by Dr. Richardson, of London, and his scientific deductions have been received on this question, as on all other matters, as incontrovertible truths. Dr. Richardson found that the difference of action of the alcohols as they ascend in the series, and as the carbon increases, is very marked. The slowness of action, the prolongation of action, step by step, from the lighter to the heavier compounds, is truth as definite as any in physiology. He observes as a singular fact, evidenced in all his experiments, that common ethylic alcohol, while it produces stupor, does not, unless it be long continued, induce tremors or convulsions, while butylic and amylic alcohol directly produced these effects. The tremors, caused by amylic alcohol are most persistent; they are called forth by the smallest excitement, and complete recovery from them, as indicated by return of natural temperature, is not attained even when the alcohol is withdrawn in a shorter interval than three days. The same author has shown that the animal temperature is more determinately reduced by the heavier alcohols, and as they are more frequently used, he supposed them to be the cause of delirium tremens and the lassitude, depression of spirits, coldness, etc., following a debauch. Dr. Richardson's deductions afford us an explanation of the fact that so few cases of delirium tremens occur in the United States at the present compared with former times.

The whiskey sold in the restaurants and shops, is in nearly every instance rectified and compounded, and contains very little alcohol. The true physiological theory perhaps is, that a certain amount of alcohol is decomposed in the system, but that a larger amount remains locked up in the brain, liver, etc., where it accumulates until it finally produces what is termed chronic alcoholism, that it may for a time, and in some slight degree arrest waste, but that in the end, by its causing the retention of effete matter in the system, it leads to degeneration. This effete matter, the urea, chlorides, etc., is retained owing to a paralysis of the nerves.

The therapeutic action of alcohol has excited an equal amount of discussion, and there exists as great a variety and divergence of opinion concerning it. It is generally conceded, however, that in those cases of acute disease in which it appears to act well, it acts as a depressant; that is, it lowers high temperature, lessens frequency of the pulse, and by arousing a transient strength, tranquilizes those nervous disturbances of the system which accompany a high degree of pyrexia. Yet there are patients who cannot tolerate its use, even in this condition, and to whom its administration is an injury.

A physician of much observation and experience assures us that whenever he discovers it on the breath of a patient suffering from typhoid or other fever, he feels assured that it is not assimilated, and is doing injury. Would this not be a good test in the healthy also? The mode of its action in the pyrexia is not clearly understood; but it is supposed that it owes its influence to an antiseptic power by which it destroys those bodies or organisms which induce a febrile state of the blood. In inflammatory conditions it is supposed to contract the arterioles through its influence on the sympathetic, and to interfere with the migration of the white corpuscles of the blood. Its action in chronic diseases is as uncertain as the modes of its administration are various and irregular. Debility is the bugbear which a class of practitioners have always before their eyes, and for which they are ever wildly prescribing, and with them alcohol, as an agent, occupies the first rank, not even second to that of iron.

Pathological effects of alcohol. The pathological changes or morbid effects of alcohol next claim our attention. These are best exemplified in the very careful and candid investigations of Dr. Dickinson, St. George's Hospital. His views are based upon an analysis of the post mortem and case books of that hospital for a period of thirty years. This comprised the particulars of the examination of the bodies of one hundred and forty-nine traders in liquors. For comparison there were taken from the same source the same number of examinations of persons otherwise and very variously employed, chosen by rule so as to afford a fair standard.

A comparison of the ages at which each class died, showed that to trade in liquor cost three and a half years of life; the alcoholic trader dying at the age of 36 8-10 years, the ordinary patient at 40 6-8 years, the shortness of life in both patients being adduced to the fact that they were hospital patients. The morbid alterations of the two series were as follows: Passing over the stomach, the changes in which are not always evident through morbid anatomy, it was shown, with regard to the liver, that in the alcoholic series there was a great and unmistakable increase of cirrhosis, which disease was found in twenty-two persons of the alcoholic to eight of the non-alcoholic series, the liability to cirrhosis being thus nearly trebled by alcoholic abuse. Fatty degeneration was also increased. In the lungs pneumonia proved to be rather less frequent with the alcoholic, but empyæmia, as distinguished from simple pleurisy, belonged especially to the alcoholic series.

The next important conclusion related to tubercle, which in every shape was most frequent in the alcoholic traders. As the relation between alcohol and tubercles has been the subject of some difference of opinion, it was shown in detail that under alcohol every kind of disease of the lungs to which the term tubercular could be applied was increased. The increase, however, was greatest where the disease was certified as truly tubercular, by the concurrence of disease of the same kind in other organs. This multiple form of disease affected thirty persons of the alcoholic series, nineteen of the non-alcoholic. Tubercle, including all kinds, affected the lungs in sixty-one persons of the alcoholic to forty-four of the non-alcoholic class. It was further shown, that in each of the following organs, the brain, the liver, the kidneys, the spleen, bowels, mesenteric glands, and peritoneum, tubercle was at least twice as common in the alcoholic as in the non-alcoholic category. The conclusion is inevitable that alcohol engenders tubercle. The brain also in the alcoholic class displayed a remarkable preponderance of inflammatory affections. Strange to say, the kidneys, which are generally supposed to be morbidly susceptible to the influence of alcohol, were found very slightly affected. Dr. Dickinson's general conclusions are thus summed up: Alcohol causes fatty infiltration and fibroid encroachment, it engenders tubercle, encourages suppuration and retards healing; it produces untimely atheroma, invites hemorrhage, and anticipates age.

The most constant fatty change, replacement by oil of the material of epithelial cells and muscular fibres, though probably nearly universal, is most noticeable in the liver, the heart, and the kidneys. The fibroid increase occurs about the vascular channels and superficial investments of the viscera, where it causes atrophy, cirrhosis, and granulation. Of this change the liver has the largest share, the lungs are often similarly but less simply affected, the change being variously complicated with or simulation of tubercle; the kidneys suffer in a more remote degree. Alcohol also causes vascular deteriorations, which are akin both to the fatty and the fibroid.

Doctor Dickinson's conclusion that alcohol engenders tubercle, is a very singular circumstance in view of the fact that whiskey is the great panacea in modern times for the arrest and cure of tuberculosis.

Having thus briefly examined the physiological and therapeutic action of alcohol and the morbid changes induced by its use, we are prepared to discuss the first four propositions named in the beginning of this paper.

From a careful study of the subject, as well as from our own experience, we are constrained to believe that alcohol possesses very limited powers of alimentation. Indeed, Subbotin maintains that it cannot be regarded in any sense as a food. He terms it a *reiz* or *geniess mittel*. Voit, whilst not entirely agreeing with him as to the mode of action of alcohol, concurs in the opinion that it is an excitant or *reiz mittel*. The experience of all wars since the invasion of Egypt by Vespasian has shown most conclusively the superiority of coffee as a dietetic agent, and its greater powers in enabling men to withstand fatigue and privation.

(TO BE CONTINUED.)

